# Algorithm-Optimized H5 Influenza mRNA Vaccine Induces Broad Immune Responses

**DOI:** 10.3390/ijms27104547

**Published:** 2026-05-19

**Authors:** Liangliang Wang, Zhengda Peng, Chenchen He, Jie Zhang, Pengju Yu, Weijin Huang, Youchun Wang, Chenyan Zhao

**Affiliations:** 1 Division of HIV/AIDS and Sex-Transmitted Virus Vaccines, Institute for Biological Product Control, National Institutes for Food and Drug Control (NIFDC), Beijing 102629, China; 2 Chinese Academy of Medical Sciences and Peking Union Medical College, Beijing 100730, China; 3 State Key Laboratory of Drug Regulatory Science, Beijing 102629, China; 4 Institute of Medical Biology, Chinese Academy of Medical Sciences and Peking Union Medical College, Kunming 650118, China

**Keywords:** H5 avian influenza virus, algorithm optimization, mRNA vaccine, broadly protective vaccine, pandemic

## Abstract

The high case fatality rate, cross-species transmission, and ongoing evolution of H5 avian influenza viruses pose an imminent threat of an influenza pandemic, particularly with the currently predominant clade 2.3.4.4b lineage. Existing seasonal influenza vaccines and licensed H5 vaccines provide limited cross-protection against H5 viruses, underscoring an urgent need for the development of broadly protective H5 vaccines. In this study, we analyzed all human-infected H5 hemagglutinin (HA) sequences using bioinformatics approaches and subsequently designed a novel H5 influenza vaccine through algorithm optimization. The predicted structure of this vaccine closely resembles that of the wild-type H5 HA trimer. In animal studies, the algorithm-optimized H5 mRNA vaccine not only induced high levels of neutralizing antibodies against multiple clade 2.3.4.4b H5 viruses but also elicited cross-neutralizing antibodies against clade 2.3.4.4 and clade 2.2.1 H5 viruses, as well as robust cellular immune responses. These findings highlight the potential of algorithm-based approaches in developing broadly protective vaccines against pandemic viruses and suggest that this vaccine candidate could serve as a strategic stockpile for preventing H5 influenza pandemics.

## 1. Introduction

Influenza A viruses remain one of the major global public health threats, causing seasonal epidemics annually and occasional pandemics. Among the numerous subtypes of influenza A viruses, H5 highly pathogenic avian influenza (HPAI) poses a concerning pandemic threat due to its high case fatality rate and expanding host range. Since the first documented human infection with H5 avian influenza in 1997, H5 has exhibited a case fatality rate exceeding 50% [1,2]. According to the World Health Organization, a cumulative total of 993 human cases of H5N1 infection and 477 deaths have been reported from 2003 to 2025 [3]. Notably, since February 2024, the H5 influenza virus of clade 2.3.4.4b has crossed the species barrier, causing outbreaks in dairy cattle in the United States, affecting over 1000 herds, and has resulted in more than 70 human infections to date [4]. Although no human-to-human transmission of H5 avian influenza has been reported, studies have demonstrated that H5 mutant strains obtained through serial passage in ferrets can transmit via aerosols among ferrets [5], indicating that H5 can acquire the capacity for airborne transmission among mammals without the need for reassortment with intermediate hosts. With further mutations of H5 avian influenza viruses, H5 has the potential to cause a global pandemic.

The most effective measure for controlling a pandemic is the development of an effective broadly protective H5 vaccine. Current seasonal influenza vaccines require lengthy production cycles and cannot provide immune protection against H5 avian influenza. The United States has approved three H5 influenza vaccines [6]; however, none of these vaccines contain the currently predominant lineage clade 2.3.4.4b [7,8], and they provide limited cross-protection. Therefore, novel technologies are needed to develop an H5 influenza vaccine capable of eliciting cross-protection. In recent years, algorithm-optimized vaccine design has demonstrated significant advantages in the development of broadly protective vaccines. A Mosaic algorithm-designed influenza live-attenuated vaccine elicited robust cross-reactive humoral and mucosal immune responses in mice and established tissue-resident memory T cells and B cells in the respiratory tract [9]. A phylogenetic algorithm-designed adenovirus-vectored coronavirus vaccine demonstrated exceptional broad-spectrum protection in mouse models, effectively preventing infection by multiple SARS-CoV-2 variants and providing protection against multiple sarbecoviruses, including SARS-CoV [10]. The EVE-Vax algorithm enables precise design of SARS-CoV-2 spike proteins with novel mutation combinations representing future antigenic evolution, providing a new approach for vaccine design against future viral strains [11]. Furthermore, the rapid production and strong immunogenicity of mRNA vaccines offer favorable conditions for developing vaccines against pandemic infectious diseases [12,13].

In this study, we employed the Epi algorithm [14] to optimize all human-infected H5 hemagglutinin (HA) sequences, generating novel candidate immunogens. The predicted structure of the algorithm-derived immunogen closely resembles that of the wild-type A/Jiangsu/NJ210/2023 (H5N1) strain HA. A/Jiangsu/NJ210/2023 (H5N1) was isolated from a human infection case in Jiangsu, China, representing a clade 2.3.4.4b H5 strain capable of causing disease in humans [15]. The mRNA vaccine developed based on the candidate immunogen could be expressed in vitro and elicited cross-neutralizing antibodies against the predominant lineage clade 2.3.4.4b H5 viruses as well as neutralizing antibodies against clade 2.3.4.4 and clade 2.2.1 viruses in mice. Furthermore, the vaccine induced robust cellular immune responses and H5 antigen-specific IgG antibody-secreting cell responses in mouse spleens.

## 2. Results

### 2.1. Design and Characterization of Algorithm-Optimized H5 Influenza Vaccine

**Figure 1:**
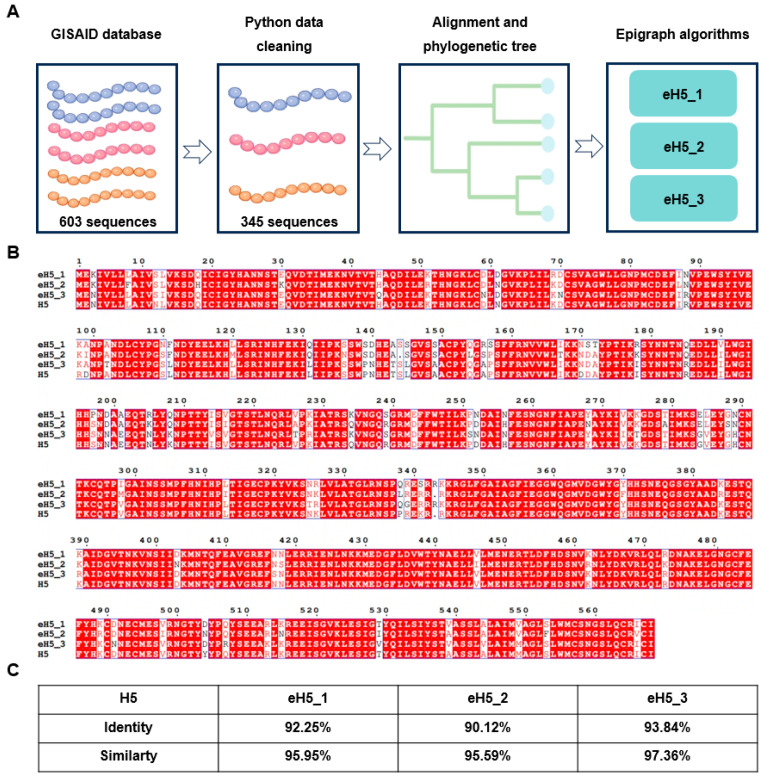
Design of algorithm-optimized H5 influenza vaccine. (**A**) Workflow of algorithm-optimized vaccine design. All human-infected H5 HA sequences were downloaded from the GISAID database, followed by data curation and multiple sequence alignment using Python. The aligned sequences were then processed using the algorithm to generate three candidate HA sequences (eH5_1, eH5_2, and eH5_3). (**B**) HA sequence alignment of H5, eH5_1, eH5_2, and eH5_3. H5, A/Jiangsu/NJ210/2023 (H5N1). (**C**) Identity and similarity of HA protein sequences between eH5_1, eH5_2, eH5_3, and H5.

To design an H5 influenza vaccine with potential broad-spectrum protection, we first downloaded all human-infected H5 HA sequences from the GISAID database. Subsequently, we performed data curation using a Python program, followed by multiple sequence alignment of the curated sequences. The aligned sequences were then optimized using the Epi algorithm to generate three candidate HA sequences (eH5_1, eH5_2, and eH5_3) (Figure 1A). A/Jiangsu/NJ210/2023 (H5N1), isolated from a human infection case in Jiangsu, China, represents a clade 2.3.4.4b H5 influenza strain capable of causing disease in humans [15]. As clade 2.3.4.4b is the currently predominant lineage of H5 influenza, we selected A/Jiangsu/NJ210/2023 (H5N1) as the control vaccine strain (hereinafter referred to as H5). Multiple sequence alignment of eH5_1, eH5_2, eH5_3, and H5 using a Python program revealed that eH5_3 shared the highest identity and similarity with H5 (Figure 1B,C). To enhance experimental comparability, eH5_3 was selected as the algorithm-designed candidate vaccine strain for subsequent experiments (hereinafter referred to as eH5).

**Figure 2:**
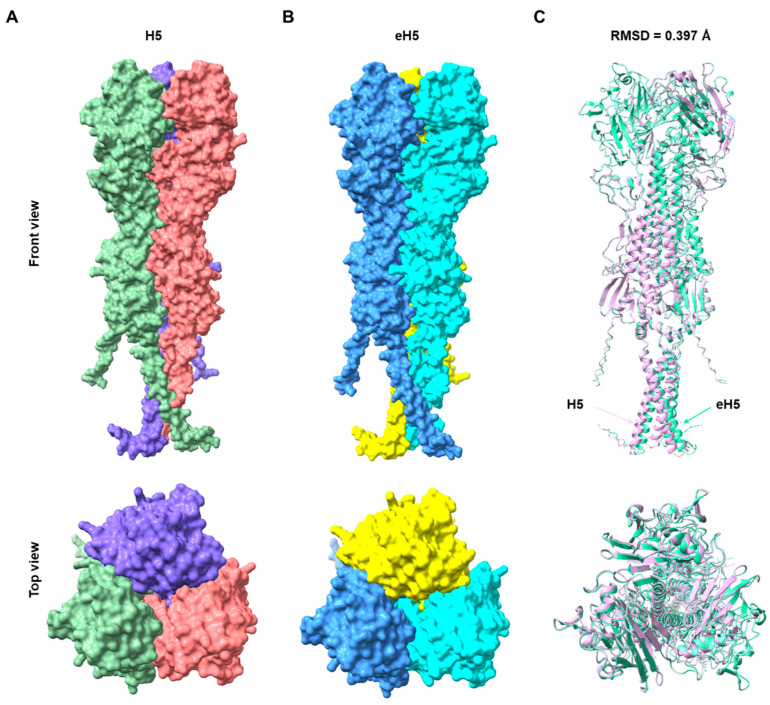
Structure prediction and structural alignment. (**A**) H5 trimer structure predicted by AlphaFold 3. (**B**) eH5 trimer structure predicted by AlphaFold 3. H5, A/Jiangsu/NJ210/2023 (H5N1). (**C**) Structural alignment of eH5 and H5.

**Figure 3:**
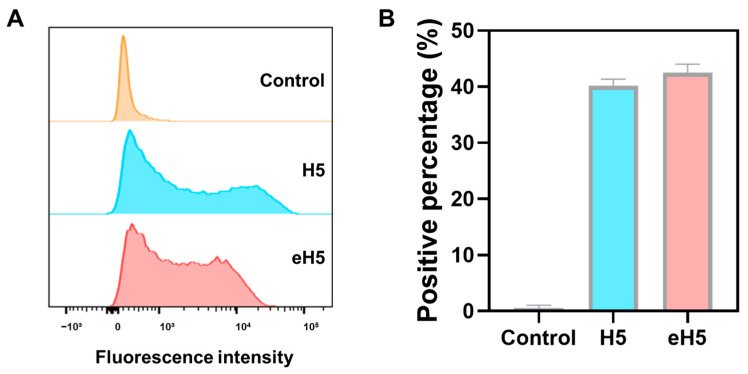
In vitro expression of algorithm-optimized H5 mRNA vaccine. mRNA with a C-terminal Flag tag was transfected into HeLa cells for 24 h. Cells were fixed, permeabilized, and incubated with fluorescent anti-Flag antibody. Representative flow cytometry histograms (**A**) and percentages of positive cells (**B**) were obtained by flow cytometry. Data are presented as mean ± SD (*n* = 3). Control, negative control.

To validate whether the algorithm-designed eH5 could form a trimer structure similar to the native structure, we predicted its structure using AlphaFold 3 [16]. As shown in Figure 2A,B, eH5 was able to form a trimer structure similar to that of H5. Structural alignment using UCSF ChimeraX (Version 1.11.1) [17] revealed that the root mean square deviation (RMSD) between eH5 and H5 was only 0.397 Å (Figure 2C). mRNA technology has demonstrated significant advantages in various infectious disease vaccines and enables rapid response to disease outbreaks. We developed mRNA vaccines encoding H5 and eH5, respectively, with a Flag tag added at the C-terminus to facilitate expression validation. Flow cytometry analysis at 24 h post-transfection of HeLa cells confirmed the correct expression of H5 and eH5 mRNA (Figure 3A,B). These data indicate that the algorithm-designed eH5 mRNA vaccine exhibits structural similarity to the native structure and can be expressed in vitro.

### 2.2. Algorithm-Optimized H5 mRNA Vaccine Induces Broad Immune Responses

**Figure 4:**
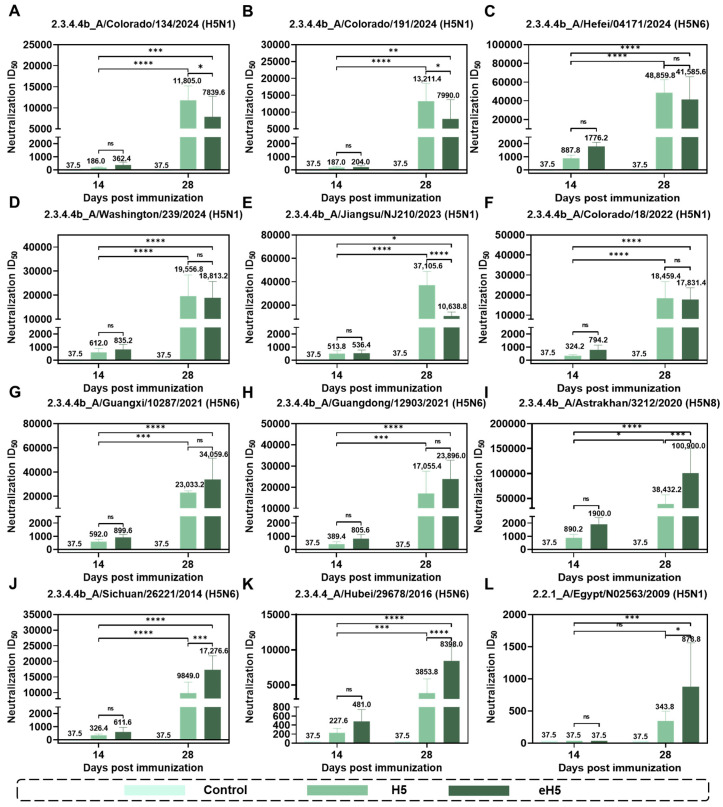
Broad neutralizing antibody responses induced by algorithm-optimized H5 mRNA vaccine. (**A**–**L**) Mice were immunized via intramuscular injection with 10 μg of algorithm-optimized H5 mRNA vaccine (eH5) or A/Jiangsu/NJ210/2023 (H5N1) mRNA vaccine (H5), with saline injection as a control. Mice received two doses at a 14-day interval, and sera were collected at 14 and 28 days after prime immunization to assess neutralizing antibody levels against A/Colorado/134/2024 (H5N1) (**A**), A/Colorado/191/2024 (H5N1) (**B**), A/Hefei/04171/2024 (H5N6) (**C**), A/Washington/239/2024 (H5N1) (**D**), A/Jiangsu/NJ210/2023 (H5N1) (**E**), A/Colorado/18/2022 (H5N1) (**F**), A/Guangxi/10287/2021 (H5N6) (**G**), A/Guangdong/12903/2021 (H5N6) (**H**), A/Astrakhan/3212/2020 (H5N8) (**I**), A/Sichuan/26221/2014 (H5N6) (**J**), A/Hubei/29578/2016 (H5N6) (**K**), and A/Egypt/N02563/2009 (H5N1) (**L**) pseudoviruses by pseudovirus neutralization assay. Data are presented as mean ± SD (*n* = 5). Values in the figure represent the mean value. (* *p* < 0.05, ** *p* < 0.01, *** *p* < 0.001, **** *p* < 0.0001, ns, not significant).

**Figure 5:**
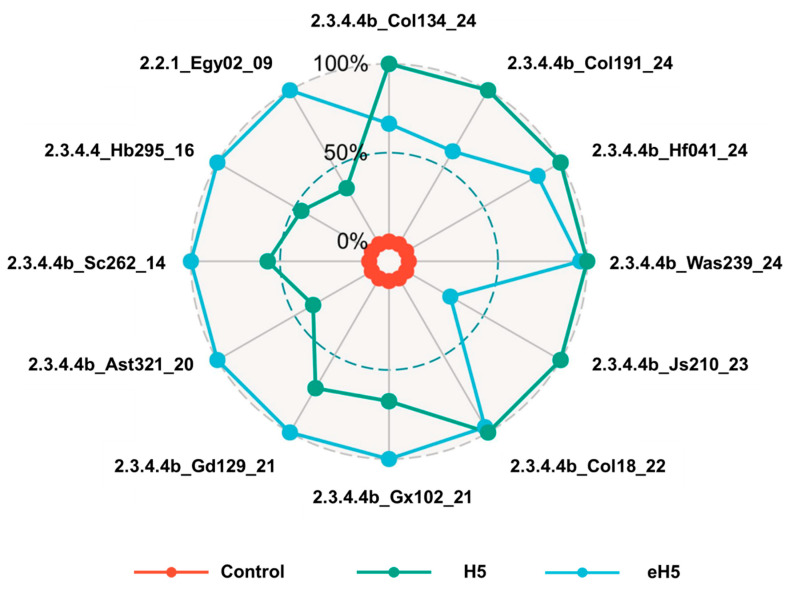
Radar plot of neutralizing antibody levels against multiple H5 influenza pseudovirus strains. The radar plot displays standardized neutralizing antibody levels against various pseudovirus strains based on pseudovirus neutralization assay results. Col134_24, A/Colorado/134/2024 (H5N1); Col191_24, A/Colorado/191/2024 (H5N1); Hf041_24, A/Hefei/04171/2024 (H5N6); Was239_24, A/Washington/239/2024 (H5N1); Js210_23, A/Jiangsu/NJ210/2023 (H5N1); Col18_22, A/Colorado/18/2022 (H5N1); Gx102_21, A/Guangxi/10287/2021 (H5N6); Gd129_21, A/Guangdong/12903/2021 (H5N6); Ast321_20, A/Astrakhan/3212/2020 (H5N8); Sc262_14, A/Sichuan/26221/2014 (H5N6); Hb295_16, A/Hubei/29678/2016 (H5N6); Egy02, A/Egypt/N02563/2009 (H5N1). Clades 2.3.4.4b, 2.3.4.4, and 2.2.1 represent H5 lineage designations.

To evaluate the humoral immune responses induced by the algorithm-optimized H5 mRNA vaccine, mice were immunized via intramuscular injection with 10 μg of algorithm-optimized H5 mRNA vaccine (eH5) or A/Jiangsu/NJ210/2023 (H5N1) mRNA vaccine (H5), with saline injection as a control. Mice received two doses at a 14-day interval, and sera were collected at 14 and 28 days after prime immunization to assess humoral immunity by pseudovirus neutralization assay and enzyme-linked immunosorbent assay (ELISA). Since clade 2.3.4.4b is the currently predominant lineage, we tested a larger number of clade 2.3.4.4b H5 circulating strains. As shown in Figure 4, compared with 14 days after prime immunization, the eH5 group induced high levels of neutralizing antibodies against 12 influenza pseudoviruses at 28 days after prime immunization, whereas the H5 group induced high levels of neutralizing antibodies against 11 influenza pseudoviruses at 28 days after prime immunization. For the H5 group, neutralizing antibody levels against the clade 2.2.1 strain A/Egypt/N02563/2009 (H5N1) showed no statistically significant difference between 14 and 28 days after prime immunization. We further analyzed the neutralizing antibody data at 28 days after prime immunization using standardized methods. As shown in Figure 5, for clade 2.3.4.4b H5 influenza strains isolated after 2022, the eH5 group induced neutralizing antibody levels similar to those of the H5 group against A/Hefei/04171/2024 (H5N6), A/Washington/239/2024 (H5N1), and A/Colorado/18/2022 (H5N1) pseudoviruses, whereas neutralizing antibody levels induced by the eH5 group against A/Colorado/134/2024 (H5N1), A/Colorado/191/2024 (H5N1), and A/Jiangsu/NJ210/2023 (H5N1) pseudoviruses were lower than those in H5-immunized mice. For clade 2.3.4.4b H5 influenza viruses from 2014 to 2021, the eH5 group induced significantly higher neutralizing antibody levels against A/Guangxi/10287/2021 (H5N6), A/Guangdong/12903/2021 (H5N6), A/Astrakhan/3212/2020 (H5N8), and A/Sichuan/26221/2014 (H5N6) pseudoviruses compared with the H5 group (Figure 5). Furthermore, for clade 2.3.4.4 and clade 2.2.1, the eH5 group induced significantly higher neutralizing antibody levels against A/Hubei/29578/2016 (H5N6) and A/Egypt/N02563/2009 (H5N1) compared with the H5 group.

To evaluate binding antibody responses, enzyme-linked immunosorbent assay (ELISA) was performed to assess HA-specific immunoglobulin G (IgG) and IgG subtypes (IgG1, IgG2a, and IgG2b) against A/Jiangsu/NJ210/2023 (H5N1) and A/Astrakhan/3212/2020 (H5N8) HA in sera collected at 14 and 28 days after prime immunization. As shown in Figure S1, both H5 and eH5 groups exhibited significantly enhanced binding antibody levels of IgG, IgG1, IgG2a, and IgG2b against A/Jiangsu/NJ210/2023 (H5N1) HA at 28 days compared with 14 days after prime immunization. For IgG binding antibodies against A/Jiangsu/NJ210/2023 (H5N1) HA, the H5 group showed higher antibody levels than the eH5 group at both 14 and 28 days after prime immunization (Figure S1C). Regarding IgG1 binding antibodies against A/Jiangsu/NJ210/2023 (H5N1) HA, the H5 group displayed higher antibody levels than the eH5 group at 28 days after prime immunization (Figure S1F). However, no significant differences were observed in binding antibody levels of other subtypes at different time points (Figure S1F,I,L). As shown in Figure S2, both H5 and eH5 groups demonstrated significantly elevated binding antibody levels of IgG, IgG1, IgG2a, and IgG2b against A/Astrakhan/3212/2020 (H5N8) HA at 28 days compared with 14 days after prime immunization. Notably, regardless of specific total IgG or IgG1, IgG2a, and IgG2b subtypes, no statistically significant differences were detected between H5 and eH5 groups (Figure S2), suggesting that binding antibody responses in mice may have reached a saturation state at this time point.

To systematically evaluate the level of functional humoral immune responses induced by the vaccines, this study further employed the Hemagglutination Inhibition (HI) assay to measure HI antibody titers against the inactivated A/Jiangsu/NJ210/2023 (H5N1) vaccine strain in serum samples collected from mice 28 days after primary immunization. The detection results are presented in Figure S3, 28 days post-primary immunization. HI antibody titers in both the wild-type H5 group and the eH5 group were above 1:40, indicating that immunization with either vaccine could elicit hemagglutination inhibition antibody responses with potential protective efficacy. Between-group comparisons revealed that the HI antibody level in the wild-type H5 group was significantly higher than that in the eH5 group (Figure S3), and this difference aligns with expectations: the sequence of the wild-type H5 antigen is fully matched to the test strain, enabling induction of higher levels of specific HI antibodies; whereas the eH5 broad-spectrum antigen needs to balance immunogenicity across different clades, resulting in slightly lower HI antibody levels against this specific strain compared to the homologous vaccine, though still meeting the protective threshold requirement.

Collectively, the algorithm-optimized H5 mRNA vaccine elicited broad neutralizing antibody responses in mice.

### 2.3. Algorithm-Optimized H5 mRNA Vaccine Elicits Robust Cellular Immune Responses in Mice

**Figure 6:**
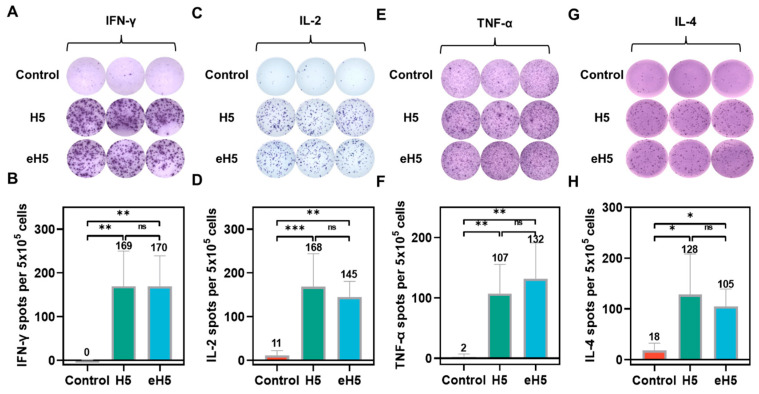
Cellular immune responses induced by algorithm-optimized H5 mRNA vaccine. (**A**–**H**) Mice were immunized via intramuscular injection with 10 μg of algorithm-optimized H5 mRNA vaccine (eH5) or A/Jiangsu/NJ210/2023 (H5N1) mRNA vaccine (H5), with saline injection as a control. Mice received two doses at a 14-day interval, and splenocytes were harvested at 28 days after prime immunization and stimulated with an HA peptide pool from A/Jiangsu/NJ210/2023 (H5N1) to assess cellular immunity by enzyme-linked immunospot (ELISpot) assay. Representative IFN-γ spot images (**A**) and quantification of IFN-γ spot-forming cells (**B**); representative IL-2 spot images (**C**) and quantification of IL-2 spot-forming cells (**D**); representative TNF-α spot images (**E**) and quantification of TNF-α spot-forming cells (**F**); representative IL-4 spot images (**G**) and quantification of IL-4 spot-forming cells (**H**). Data are presented as mean ± SD (*n* = 5). Values in the figure represent the mean value. (* *p* < 0.05, ** *p* < 0.01, *** *p* < 0.001, ns, not significant).

To evaluate cellular immune responses induced by the algorithm-optimized H5 mRNA vaccine, spleens were harvested at 28 days after prime immunization following the immunization protocol described above, and cellular immunity was assessed by enzyme-linked immunospot (ELISpot) assay. Upon in vitro stimulation with an HA peptide pool from A/Jiangsu/NJ210/2023 (H5N1), both H5 and eH5 groups generated higher numbers of interferon-γ (IFN-γ), interleukin-2 (IL-2), tumor necrosis factor-α (TNF-α), and interleukin-4 (IL-4) spot-forming cells compared with the control group (Figure 6). Notably, even when stimulated with the HA peptide pool from A/Jiangsu/NJ210/2023 (H5N1), the eH5 group elicited comparable numbers of IFN-γ (Figure 6A,B), IL-2 (Figure 6C,D), TNF-α (Figure 6E,F), and IL-4 spot-forming cells (Figure 6G,H) to those of the H5 group. These findings demonstrate that the algorithm-optimized H5 mRNA vaccine induces robust cellular immune responses in mice.

### 2.4. Algorithm-Optimized H5 mRNA Vaccine Elicits Robust H5 Antigen-Specific IgG Antibody-Secreting Cell Responses in Mice

**Figure 7:**
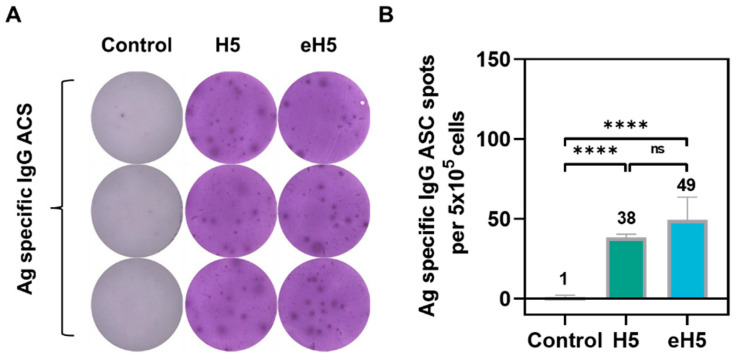
Antigen-specific IgG antibody-secreting cells (ASCs) in splenocytes from mice immunized with algorithm-optimized H5 mRNA vaccine. (**A**,**B**) Mice were immunized via intramuscular injection with 10 μg of algorithm-optimized H5 mRNA vaccine (eH5) or A/Jiangsu/NJ210/2023 (H5N1) mRNA vaccine (H5). Mice received two doses at a 14-day interval, and splenocytes were harvested at 28 days after prime immunization and incubated with A/Jiangsu/NJ210/2023 (H5N1) HA protein in vitro for 24 h, followed by detection of antigen-specific IgG ASCs by enzyme-linked immunospot (ELISpot) assay. Representative antigen-specific IgG ASC spot images (**A**) and quantification of antigen-specific IgG ASC spot-forming cells (**B**). Data are presented as mean ± SD (*n* = 5). Values in the figure represent the mean value. (**** *p* < 0.0001, ns, not significant).

To evaluate antigen-specific IgG antibody-secreting cells (ASCs) induced by the algorithm-optimized H5 mRNA vaccine, spleens were harvested at 28 days after prime immunization following the immunization protocol described above, and the number of H5 antigen-specific IgG ASCs was assessed by enzyme-linked immunospot (ELISpot) assay. As shown in Figure 7A,B, both H5 and eH5 groups generated significantly higher numbers of H5 antigen-specific ASCs compared with the control group. Notably, even when the coating protein was A/Jiangsu/NJ210/2023 (H5N1) HA, the eH5 group elicited comparable numbers of antigen-specific ASCs to those of the H5 group (Figure 7A,B). These findings demonstrate that the algorithm-optimized H5 mRNA vaccine induces robust H5 antigen-specific IgG antibody-secreting cell responses in mice.

### 2.5. Proteomic Reprogramming in Mouse Spleen Induced by Algorithm-Optimized H5 mRNA Vaccine

**Figure 8:**
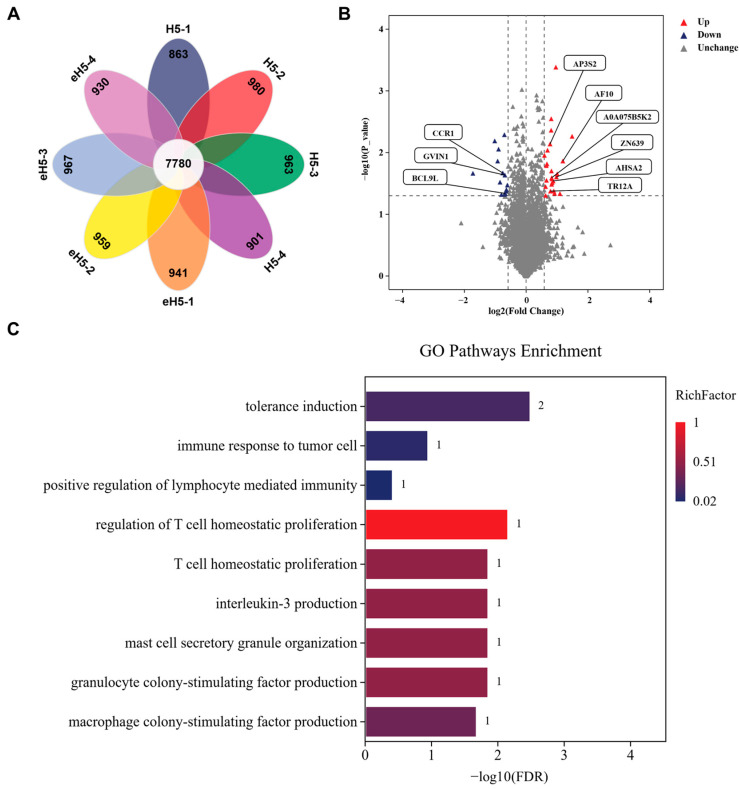
Proteomic profiles of mouse spleen after immunization with algorithm-optimized H5 mRNA vaccine. Mice were immunized via intramuscular injection with 10 μg of algorithm-optimized H5 mRNA vaccine (eH5) or A/Jiangsu/NJ210/2023 (H5N1) mRNA vaccine (H5). Mice received two doses at a 14-day interval, and spleens were harvested at 28 days after prime immunization for proteomic sequencing. (**A**) Overlap of identified proteins across different samples. The number in the center of the petal diagram represents the count of shared proteins identified across all samples, while numbers around the petals indicate the counts of unique proteins identified in each sample. (**B**) Volcano plot of differentially expressed proteins between groups. Red represents upregulated proteins, blue represents downregulated proteins, and gray represents non-differentially expressed proteins. (**C**) Gene Ontology (GO) pathway enrichment bar chart.

To evaluate whether proteomic profiles in the spleen differed between eH5 and H5 groups after immunization, spleens were harvested at 28 days after prime immunization following the same immunization protocol for proteomic sequencing. As shown in Figure 8A, a total of 7780 shared proteins were detected in both eH5 and H5 groups, demonstrating high overall similarity of proteomes between the two groups (>95%), which ensured the reliability of subsequent differential analysis. The 4 replicates in the eH5 group shared a total of 8098 proteins, while the 4 replicates in the H5 group shared 8090 proteins. The protein uniquely detected in all eH5 samples was Q8R216, which is associated with antigen presentation pathway activation. There were 2 proteins uniquely detected in all H5 samples, namely Q7TNM2 and Q8BXJ9, both of which are basal functional proteins not involved in immune-related regulatory pathways. Compared with the H5 group, proteins promoting B cell activation (A0A075B5K2, ZN639, and AF10) and proteins facilitating antigen presentation and T cell help (AP3S2, AHSA2, and TR12A) were significantly upregulated in the eH5 group (Figure 8B), while proteins associated with immunosuppressive pathways (CCR1, GVIN1, and BCL9L) were markedly downregulated (Figure 8B). Figure 8C presents the Gene Ontology (GO) biological process enrichment analysis of differentially expressed proteins. Tolerance induction emerged as the most significantly enriched pathway among all enriched terms. Notably, regulation of T cell homeostatic proliferation, T cell homeostatic proliferation, and positive regulation of lymphocyte-mediated immunity were concurrently and significantly enriched, with the regulation of T cell homeostatic proliferation pathway exhibiting a fold enrichment of 1.0—indicating that all annotated proteins within this pathway were differentially expressed. These findings suggest that the algorithm-optimized broad-spectrum H5 mRNA vaccine may achieve enhanced breadth through modulation of T cell homeostatic maintenance and expansion. Furthermore, pathways associated with interleukin-3 (IL-3) production, granulocyte colony-stimulating factor (G-CSF) production, macrophage colony-stimulating factor (M-CSF) production, and mast cell secretory granule organization were all significantly enriched. This implies that the algorithm-optimized vaccine may potentiate nonspecific clearance of heterologous strains by upregulating the secretion of multiple regulatory factors, thereby recruiting and activating innate immune effector cells including macrophages, granulocytes, and mast cells. Collectively, these results delineate, at the molecular level, the regulatory mechanisms by which the algorithm-optimized broad-spectrum H5 mRNA vaccine enhances immune responses, providing direct omics evidence for its cross-clade broad-spectrum efficacy.

## 3. Discussion

The cross-species transmission of H5 avian influenza and its continuous mutation to acquire human-to-human transmission capability have raised significant global public health concerns [18,19,20]. Clade 2.3.4.4b H5, as the currently predominant circulating lineage, has caused outbreaks in dairy cattle in the United States and resulted in human infections [21]. Therefore, there is an urgent need to develop a broadly protective H5 influenza vaccine, particularly against clade 2.3.4.4b H5. In this study, the H5 mRNA vaccine developed using the Epi algorithm induced cross-neutralizing antibody responses against multiple clade 2.3.4.4b strains as well as neutralizing antibody responses against clade 2.3.4.4 and clade 2.2.1 H5 viruses in mice. Furthermore, this algorithm-optimized H5 mRNA vaccine elicited robust cellular immune responses in mice.

In this study, the algorithm-optimized H5 mRNA vaccine elicited broader neutralizing antibody responses compared with the wild-type H5 vaccine, which is consistent with previous studies on algorithm-optimized H1 and H9 influenza vaccines [22,23]. Second, even when splenocytes were stimulated with overlapping peptides from wild-type A/Jiangsu/NJ210/2023 (H5N1) HA, the algorithm-optimized H5 mRNA vaccine still elicited high levels of cellular immune responses comparable to those induced by the wild-type H5 vaccine. Notably, cellular immune responses are positively correlated with vaccine protection efficacy and contribute to the establishment of cross-reactive immunity [24,25,26]. Furthermore, regarding binding antibodies, no significant differences were observed between the algorithm-optimized H5 mRNA vaccine and the wild-type H5 mRNA vaccine. This may be because binding antibodies only need to recognize any epitope on the antigen surface rather than neutralizing epitopes, and as long as antigen expression reaches a certain level, binding antibodies can be produced and secreted.

In this study, quantitative proteomic analysis of spleen tissues from immunized mice provides systematic molecular-level support for the superior broad-spectrum immunogenicity of the algorithm-optimized H5 mRNA vaccine compared to the wild-type H5 mRNA vaccine. Our results demonstrate that the optimized vaccine exerts its function by positively regulating core immune activation pathways, including B cell activation, antigen presentation, and T cell homeostatic proliferation, while simultaneously downregulating the expression of immunosuppression-related proteins. These findings offer a proteomic-level mechanistic explanation for its cross-clade broad protective efficacy from the perspective of synergistic modulation of adaptive and innate immunity.

Although our study has identified core differentially regulated pathways and key candidate proteins between the two vaccine groups, current findings remain at the level of correlative molecular characterization. Further functional experiments are therefore required to validate the causal association between these regulatory features and the broad protective efficacy of the vaccine. For subsequent investigations, the transcriptional and expression levels of core differentially expressed proteins can first be verified in samples from immunized mice using quantitative real-time PCR (qPCR) and Western blotting (WB) to confirm the reliability of the proteomic analysis results. On this basis, for key candidate molecules involved in pathways such as antigen presentation and immunosuppression regulation, in vitro gene knockout/overexpression cell models combined with dendritic cell antigen presentation efficiency assays and T cell activation and proliferation experiments can be performed to preliminarily elucidate the immunoregulatory functions of these candidate molecules. Furthermore, immune cell-specific conditional knockout mouse models targeting the corresponding genes can be constructed to evaluate in vivo how differential expression of key molecules affects the breadth of vaccine-induced neutralizing antibodies, diversity of T cell responses, and protective efficacy against heterologous viral challenge. These studies will ultimately identify core functional targets that regulate the broad immunogenicity of mRNA vaccines, providing actionable molecular directions for sequence design optimization and immunogenicity improvement of future broad-spectrum mRNA influenza vaccines.

This study has several limitations. First, due to the highly pathogenic nature of H5 avian influenza virus, which requires high-containment biosafety level laboratories for infection experiments, live virus challenge studies in mice were not conducted in this study. Second, the mechanisms underlying the broadly protective immunity induced by the algorithm-optimized vaccine were not thoroughly investigated in this study. Third, the impact of pre-existing immunity [27,28] on vaccine responses was not evaluated. Therefore, future studies will investigate the effects of pre-existing immunity, conduct live virus challenge experiments in high-containment biosafety facilities, and elucidate the mechanisms underlying broadly protective immunity.

In conclusion, the algorithm-optimized H5 mRNA vaccine developed in this study elicited high levels of neutralizing antibodies against multiple clade 2.3.4.4b H5 strains as well as high antibody levels against clade 2.3.4.4 and clade 2.2.1 H5 viruses in mice, and induced robust cellular immune responses. These findings highlight the critical role of algorithm-based models in the design of broadly protective vaccines against pandemic pathogens and support the potential of this vaccine as a stockpiled candidate for pandemic preparedness against H5 avian influenza.

## 4. Experimental Section

### 4.1. Materials

Dulbecco’s modified Eagle medium (DMEM), penicillin-streptomycin (PS), and RPMI-1640 medium were obtained from Gibco (Grand Island, NY, USA). Fetal bovine serum (FBS) was sourced from TransGen Biotech (Beijing, China). Phosphate-buffered saline (PBS) and 4% paraformaldehyde fixation solution were acquired from Biosharp (Hefei, China). Neuraminidase, TPCK-treated trypsin, and bovine serum albumin (BSA) were obtained from Sigma-Aldrich (St. Louis, MO, USA). Anti-Flag tag antibody, goat anti-mouse IgG antibody, and HA protein from A/Astrakhan/3212/2020 were obtained from Sino Biological (Beijing, China). The inactivated A/Jiangsu/NJ210/2023 (H5N1) vaccine strain was provided by Sinovac Biotech Co., Ltd. (Beijing, China). Directly labeled anti-Flag tag antibody was purchased from BioLegend (San Diego, CA, USA). CY3-conjugated goat anti-mouse secondary antibody was acquired from Servicebio (Wuhan, China). Goat anti-mouse IgG1, IgG2a, and IgG2b antibodies were obtained from Proteintech (Wuhan, China). Fixation and permeabilization solution was obtained from BD Biosciences (San Jose, CA, USA). The G*ΔG-VSV pseudotyped virus was maintained in our laboratory. jetPRIME transfection reagent was purchased from Polyplus (Strasbourg, France). Luciferase assay reagent and Nano-Glo^®^ Luciferase substrate were sourced from Promega (Madison, WI, USA). Single-component chromogenic substrate was obtained from Biopanda (Belfast, UK). Stop solution was purchased from Solarbio (Beijing, China). ELISpot kits for IFN-γ, IL-2, TNF-α, and IL-4 were obtained from Mabtech (Stockholm, Sweden).

### 4.2. Studies Involving Animals

Six- to eight-week-old female BALB/c mice were sourced from Beijing Vital River Laboratory Animal Technology Co., Ltd. (Beijing, China). The experimental protocols involving animals were reviewed and approved by the Laboratory Animal Ethics Committee of the National Institutes for Food and Drug Control (protocol number: 2025(B)018). All procedures complied with the principles of animal protection, welfare, and ethics.

### 4.3. Cell Line

Human embryonic kidney 293T (HEK-293T), Madin-Darby canine kidney (MDCK), and HeLa cell lines were propagated under standard conditions (37 °C, 5% CO_2_) in DMEM growth medium containing 10% fetal bovine serum and 1% penicillin-streptomycin.

### 4.4. Algorithm-Based Design of H5 Influenza Vaccine

An Epi-based computational approach was utilized for H5 vaccine antigen design. In summary, hemagglutinin (HA) sequences from human H5 influenza isolates were downloaded from GISAID. After data preprocessing and redundancy removal with Python (v3.12, Centrum Wiskunde & Informatica), multiple sequence alignment was conducted using the Clustal Omega package (Version 1.2.2) within the Python environment. The aligned H5 HA sequences were uploaded to the public Epi algorithm server (https://www.hiv.lanl.gov/content/sequence/EPIGRAPH/epigraph.html, accessed on 1 March 2025) for antigen design. The optimization pipeline was executed with the following parameter settings: (1) Input type was set to Aligned sequence mode; (2) Epitope length was defined as 9 amino acids; (3) The number of output vaccine candidate sequences was set to 3; (4) The number of iterations was set to 1, with a random seed of 0 to ensure result reproducibility; (5)The number of iterative optimization steps was set to 0; (6) Rare epitopes with an occurrence frequency ≤ 2 were filtered out from the dataset. Three optimized HA candidate sequences were automatically generated upon completion of the run. Subsequently, sequence identity and similarity between the designed candidates and the reference strain A/Jiangsu/NJ210/2023 (H5N1) HA were determined through Clustal Omega alignment.

### 4.5. Structural Prediction and Comparison

Trimeric structures of the candidate sequences and the A/Jiangsu/NJ210/2023 (H5N1) HA were predicted using AlphaFold 3 [16]. Structural alignment of the predicted models was performed with UCSF ChimeraX [17], and the root-mean-square deviation (RMSD) was calculated to quantify structural differences.

### 4.6. Preparation of mRNA-Loaded Lipid Nanoparticles

mRNA-LNP formulation was performed following established protocols [29]. In summary, plasmid sequences encoding HA with C-terminal Flag tags were synthesized by General Biosystems (Chuzhou, China) and subsequently linearized through enzymatic digestion. Linearized plasmids served as templates for in vitro transcription using a T7 RNA polymerase kit (E122-U025, Novoprotein, Shanghai, China). To produce modified mRNA, UTP was substituted with N1-methylpseudouridine-5′-triphosphate during the transcription reaction. Transcripts were capped using a capping kit (M082-01B, Novoprotein), precipitated overnight, pelleted by centrifugation, and resuspended in nuclease-free water. For LNP formulation, mRNA in 50 mM citrate buffer was mixed with lipids at a 1:3 ratio (aqueous:organic) using a microfluidic mixing system (NanoAssemblr Spark, Precision Nanosystems, Vancouver, BC, Canada). The resulting mRNA-LNPs were maintained at 4 °C.

### 4.7. Assessment of mRNA Expression via Flow Cytometry

HeLa cells were transfected with mRNA and harvested 24 h post-transfection. Cells were dissociated with trypsin, washed with PBS, and subjected to fixation and permeabilization using a commercial kit (catalog number 554714, BD) for 25 min. For detection, cells were stained with a directly labeled anti-Flag tag antibody (catalog number 637321, BioLegend) for 30 min. After washing, the samples were analyzed on a FACSCanto II flow cytometer (Becton, Dickinson and Company, Franklin Lakes, NJ, USA). Data analysis was performed using FlowJo software (version v10.8.1, Tree Star).

### 4.8. Immunization and Sample Collection

A two-dose immunization schedule was employed. Mice received intramuscular injections of 10 μg of algorithm-optimized H5 mRNA vaccine or A/Jiangsu/NJ210/2023 (H5N1) mRNA vaccine at 14-day intervals. The control group was administered an equal volume of saline intramuscularly. For humoral immune response evaluation, serum samples were obtained on days 14 and 28 following the prime vaccination and analyzed for binding and neutralizing antibodies. Cellular immune responses and splenic proteomic profiling were performed using splenocytes harvested at 28 days post-priming.

### 4.9. Generation and Titration of VSV-Based Influenza Pseudoviruses

VSV-based pseudoviruses displaying influenza HA were produced according to established protocols. The HA gene was codon-optimized for mammalian cells, with a Kozak sequence incorporated at the 5′ terminus. The construct was synthesized and subcloned into the pCDNA3.1(+) vector by General Biosystems Co., Ltd. HEK-293T cells grown to 80% confluence in T75 flasks were transfected with 30 μg of HA plasmid using jetPRIME reagent (101000046, Polyplus, Strasbourg, France). Concurrently, G*ΔG-VSV was added at a multiplicity of 3 × 10^4^ TCID50/mL, and the culture was maintained for 6 h. Following three washes with PBS supplemented with 1% FBS, the cells were treated with 7 mU/mL neuraminidase (N2876-625UN, Sigma-Aldrich) for 24 h. The supernatant was collected, treated with 40 μg/mL TPCK-treated trypsin (T1426-500, Sigma-Aldrich) at 37 °C for 30 min, clarified by centrifugation at 4000 rpm for 10 min, filtered, and stored at −80 °C.

For titer determination, pseudoviruses were serially diluted 3-fold and used to infect MDCK cells (3 × 10^5^ cells/mL) for 24 h. After removing the supernatant, luciferase activity was measured using a luciferase assay system (E2620, Promega) with 2 min of shaking. Luminescence was quantified using a PerkinElmer EnSight(Waltham, MA, USA) multimode plate reader. TCID50 values were calculated based on the luciferase signal intensities.

### 4.10. Pseudovirus-Based Neutralization Assay

Heat-inactivated immune sera (56 °C, 30 min) were serially diluted 3-fold in 96-well plates and mixed with 50 μL of influenza pseudovirus at 1.3 × 10^4^ TCID50/mL. Following a 1 h incubation, the supernatant was removed, and 50 μL of luciferase assay substrate (E2620, Promega) was added with 2 min of shaking. Luminescence was quantified using a PerkinElmer EnSight multimode plate reader. The serum ID50, defined as the dilution that achieved 50% inhibition of pseudovirus infection, was determined.

### 4.11. Enzyme-Linked Immunosorbent Assay (ELISA)

ELISA was performed to assess the binding activity of immune sera. Microtiter plates were coated overnight at 4 °C with 1 μg/mL HA antigens from A/Jiangsu/NJ210/2023 (H5N1) or A/Astrakhan/3212/2020 (H5N8) (40932-V08H, Sino Biological). Non-specific binding sites were blocked with 5% BSA (B2064, Sigma-Aldrich) in PBST (0.05% Tween-20 in PBS) for 2 h at room temperature. After three PBST washes, serial dilutions of serum samples were added and incubated for 1 h at room temperature. The plates were washed three times and subsequently incubated with HRP-conjugated secondary antibodies against mouse IgG (SSA007, Sino Biological), IgG1 (SA00012-1, Proteintech), IgG2a (SA00012-2, Proteintech), or IgG2b (SA00012-3, Proteintech) for 1 h at room temperature. Following five washes, TMB substrate (TMB-S-001, Biopanda) was added for 15 min, and the colorimetric reaction was terminated with stop solution (C1058, Solarbio). Absorbance was read at 450 nm on a SpectraMax M5 microplate reader (Molecular Devices, San Jose, CA, USA). Endpoint titers were calculated as the reciprocal of the highest serum dilution with OD > 2.5-fold over background. Samples with values below the detection limit were recorded as half of the detection threshold. Recombinant HA protein from A/Jiangsu/NJ210/2023 (H5N1), containing the ectodomain, was produced and purified by Sino Biological.

### 4.12. Hemagglutination Inhibition Test

To quantify serum functional antibody titers, hemagglutination inhibition (HI) assays were performed on serum samples harvested from mice at 28 days post-primary immunization. In brief, serum samples were first pretreated with receptor-destroying enzyme (RDE) for 18 h at 37 °C to remove non-specific hemagglutination inhibitors, followed by heat inactivation at 56 °C for 30 min to eliminate residual complement activity. Sera were then subjected to 2-fold serial dilutions in 25 μL of phosphate-buffered saline (PBS) per well of a 96-well V-bottom plate, before the addition of 25 μL influenza virus working solution adjusted to contain 4 hemagglutinating units. After a 45 min incubation at room temperature to allow antibody-virus binding, 50 μL of 1% fresh chicken red blood cell suspension was added to each well. The HI titer was defined as the reciprocal of the highest serum dilution that completely abrogated hemagglutination after 30 min.

### 4.13. Enzyme-Linked Immunospot (ELISPOT) Assay

T cell and B cell immune responses in immunized mice were assessed by ELISPOT. For T cell cytokine detection, pre-coated ELISPOT kits for IFN-γ (3321-4APW-10, Mabtech), IL-2 (3441-4APW-10, Mabtech), TNF-α (3511-4APW-10, Mabtech), and IL-4 (3311-4APW-10, Mabtech) were employed. Plates were washed with PBS and activated with RPMI-1640 medium supplemented with 10% FBS (11875093, Gibco) for 30 min at room temperature. Splenocytes were plated at 5 × 10^5^ cells/well and stimulated with an overlapping HA peptide pool from A/Jiangsu/NJ210/2023 (H5N1) at 10 μg/mL. Incubation times were 24 h for IFN-γ, IL-2, and TNF-α, and 48 h for IL-4. After washing, biotinylated detection antibodies were added for 2 h at room temperature, followed by ALP-conjugated streptavidin for 1 h. Spots were developed with substrate solution for ~15 min, and the reaction was terminated with tap water. Plates were air-dried and scanned using a CTL-ImmunoSpot^®^ S6 reader (Cellular Technology Limited, Shaker Heights, OH, USA). The HA peptide library consisted of 15-amino acid peptides overlapping by 11 residues, synthesized by Sangon Biotech Co., Ltd.

B cell ELISPOT was performed to quantify antigen-specific IgG-secreting cells. Plates (MSIPS4510, Merck-Millipore, Burlington, MA, USA) were coated overnight with A/Jiangsu/NJ210/2023 (H5N1) HA protein at 10 μg/mL. After PBS washing, splenocytes (5 × 10^5^ cells/well) were incubated for 24 h. Following washing, anti-mouse IgG detection antibody (3825-2A, Mabtech) was added for 2 h at room temperature. Remaining steps followed the protocol described above.

### 4.14. Mass Spectrometry-Based Proteomic Profiling

Mouse spleen tissues were collected 14 days after the second immunization. Tissues were homogenized using an MP FastPrep-24 homogenizer (MP Biomedicals, Santa Ana, CA, USA), followed by lysis with SDC buffer (5% sodium deoxycholate, 100 mM Tris-HCl, pH 8.5). The lysate was subjected to sonication and boiled for 15 min for protein denaturation. After centrifugation at 14,000× *g* for 40 min at 4 °C, the supernatant was harvested. Total protein concentration was quantified using the Bicinchoninic Acid (BCA) Protein Assay Kit, and protein extraction quality was verified via 4–20% SDS-PAGE electrophoresis followed by Coomassie Brilliant Blue R-250 staining. An equal amount of protein from each sample was pooled to prepare quality control (QC) samples for subsequent processing.

For protein digestion, dithiothreitol (DTT) was added to protein extracts at a final concentration of 10 mM to reduce disulfide bonds, with incubation at 37 °C for 1.5 h. Iodoacetamide (IAA) was then added to a final concentration of 20 mM to alkylate free cysteine residues, and the mixture was incubated at room temperature for 30 min in the dark. Sequencing-grade trypsin was added at a trypsin-to-protein mass ratio of 1:50, and digestion was performed at 37 °C overnight (16–18 h). The resulting peptides were desalted using MCX solid-phase extraction cartridges, concentrated via vacuum centrifugation, and reconstituted in 20 μL of 0.1% (*v*/*v*) formic acid aqueous solution. Indexed Retention Time (iRT) calibration peptides were spiked into each sample before mass spectrometry analysis.

Chromatographic separation was performed on a Vanquish Neo UHPLC system (Thermo Fisher Scientific, Waltham, MA, USA), and mass spectrometry data were acquired in DIA mode using an Orbitrap^TM^ Astral^TM^ mass spectrometer (Thermo Fisher Scientific). Mass spectrometry data processing and bioinformatic analysis were performed by Shanghai APTBIO Co., Ltd.

### 4.15. Statistical Analysis

All statistical analyses were conducted using GraphPad Prism 8.0 (GraphPad Software, Inc., San Diego, CA, USA). Group comparisons were performed using analysis of variance (ANOVA) with post hoc tests for multiple comparisons. Significance levels were indicated as: * *p* < 0.05; ** *p* < 0.01; *** *p* < 0.001; **** *p* < 0.0001; ns, not significant.

## Data Availability

All relevant data to the study are included in the published article or uploaded as supplemental information. The supplemental information is available free of charge.

## Supplementary Materials

The following supporting information can be downloaded at: https://www.mdpi.com/article/10.3390/ijms27104547/s1.
