# Supplementary material for: Algorithm-Optimized H5 Influenza mRNA Vaccine Induces Broad Immune Responses
Source: Int J Mol Sci. 2026 May 19;27(10):4547. doi: 10.3390/ijms27104547 (PMC13207390; doi:10.3390/ijms27104547)
Supplement: Supplementary file 1 [file ijms-27-04547-s001.zip › ijms-4252472-supplementary.pdf]

Supplemental Information

Algorithm-Optimized H5 Influenza mRNA Vaccine Induces Broad Immune Responses

Liangliang Wang[1, 2, 3 ‡], Zhengda Peng[1, 3 ‡], Chenchen He[1, 3], Jie Zhang[1, 3], Pengju Yu[1, 3], Youchun Wang[2, 4, *], Weijin Huang[1, 2, 3 *], Chenyan Zhao[1, 3 *]

[1] Division of HIV/AIDS and Sex-transmitted Virus Vaccines, Institute for Biological Product Control, National Institutes for Food and Drug Control (NIFDC), Beijing 102629, China

[2] Chinese Academy of Medical Sciences & Peking Union Medical College, Beijing 100730, China

[3] State Key Laboratory of Drug Regulatory Science, Beijing 102629, China

[4] Institute of Medical Biology, Chinese Academy of Medical Sciences &Peking Union Medical College, Kunming 650118, China

‡ These authors equally contributed to this work.

[*]Corresponding Author

Chenyan Zhao [*]: zcy1210@163.com; (Lead Contact)

Youchun Wang [*]: wangyc@imbcams.com.cn;

Weijin Huang [*]: huangweijin@nifdc.org.cn

SUPPLEMENTAL FIGURES

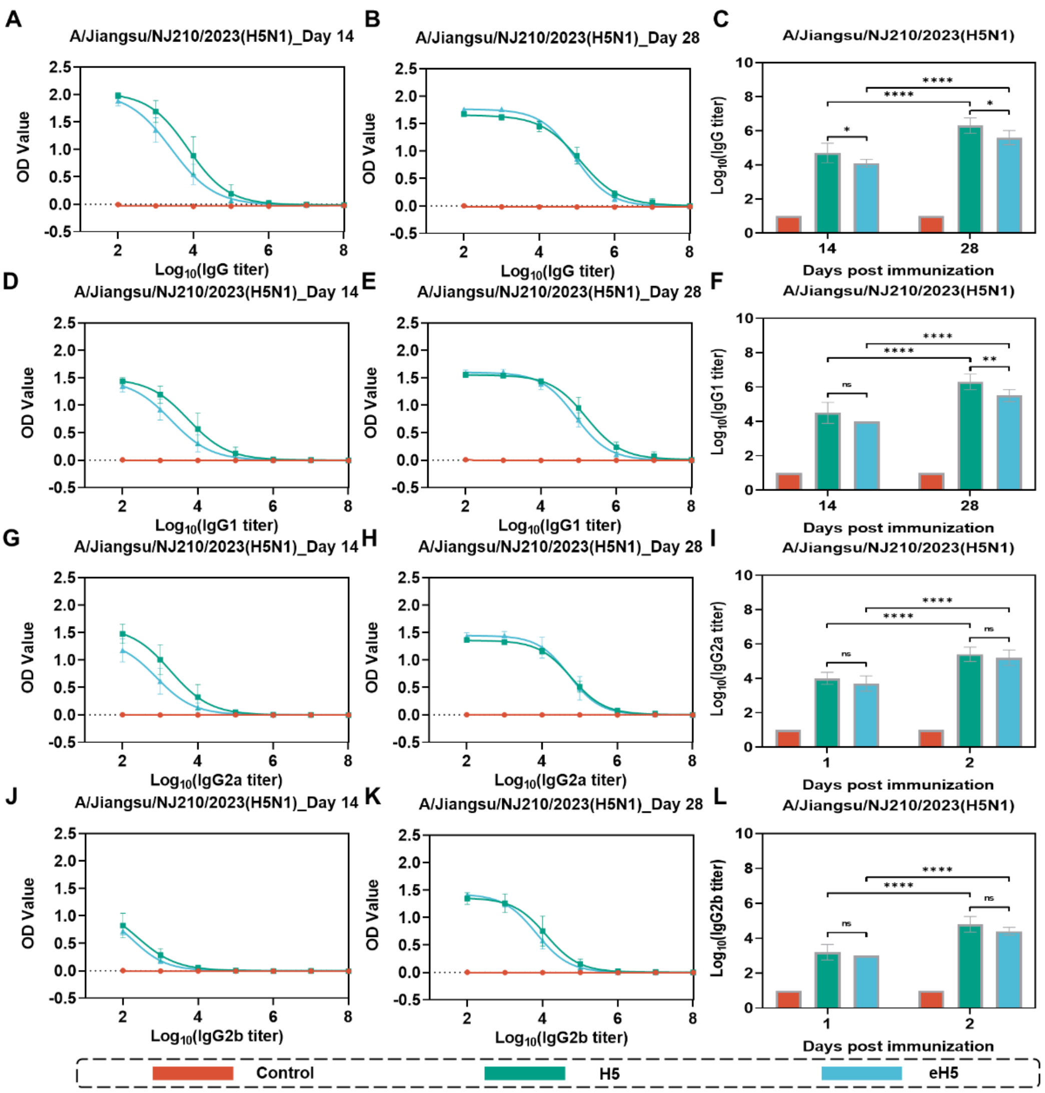

Figure S1. Binding antibody levels against A/Jiangsu/NJ210/2023 (H5N1) HA. (A-L) Mice were immunized via intramuscular injection with 10 μg of algorithm-optimized H5 mRNA vaccine (eH5) or A/Jiangsu/NJ210/2023 (H5N1) mRNA vaccine (H5). A control group received saline injection. Two doses were administered at a 14-day interval. Serum samples were collected at 14 and 28 days post-prime immunization, and binding antibody levels were evaluated by ELISA. (A and B) Serum dilution-dependent IgG binding curves to A/Jiangsu/NJ210/2023 (H5N1) HA; (C) IgG binding antibody titers. (D and E) Serum dilution-dependent IgG1 binding curves; (F) IgG1 binding antibody titers. (G and H) Serum dilution-dependent IgG2a binding curves; (I) IgG2a binding antibody titers. (J and K) Serum dilution-dependent IgG2b binding

curves; (L) IgG2b binding antibody titers. Data are presented as mean ± SD (n = 5). *$p < 0.05$, **$p < 0.01$, ***$p < 0.001$, ****$p < 0.0001$; ns, not significant.

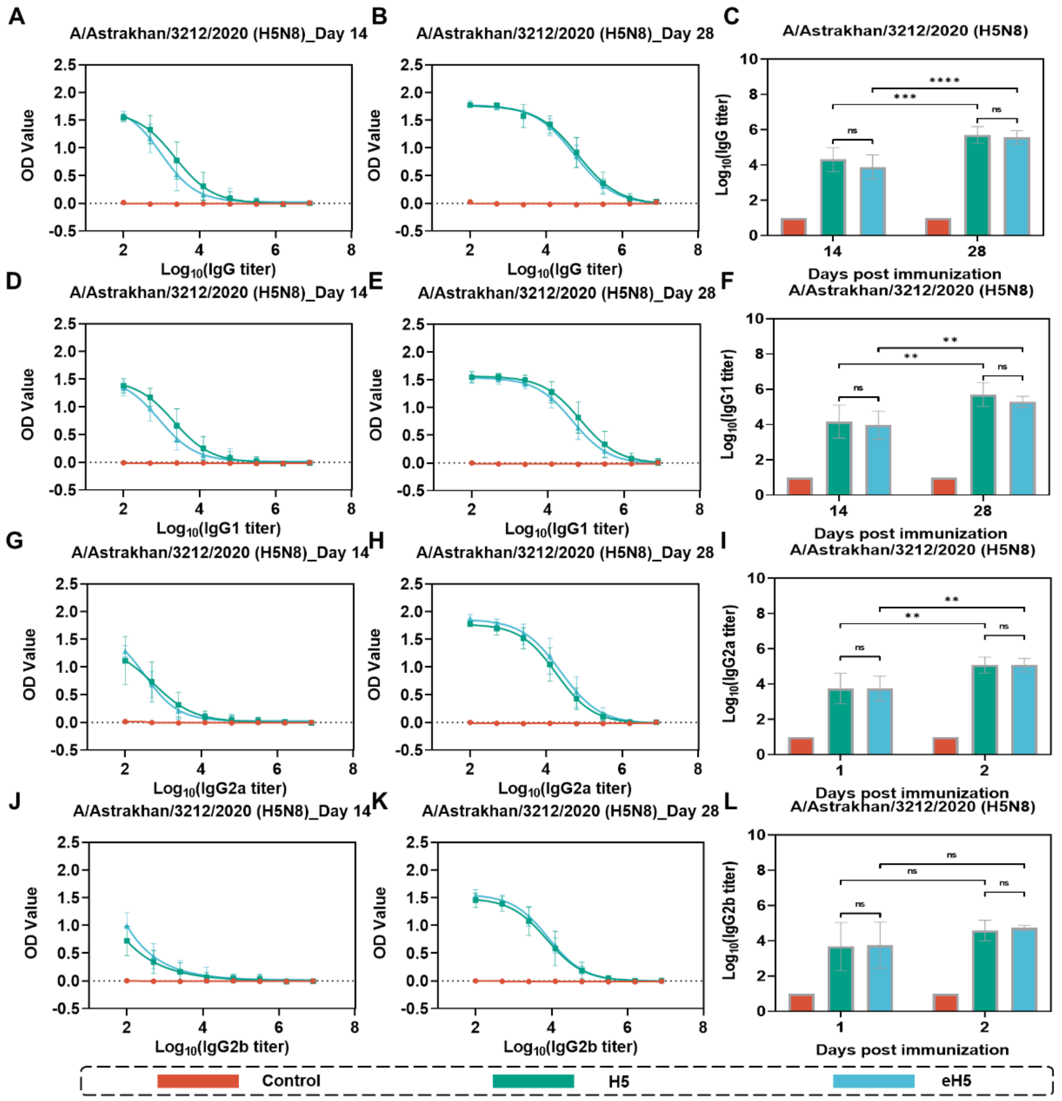

Figure S2. Binding antibody levels against A/Astrakhan/3212/2020 (H5N8) HA. (A-L) Mice were immunized intramuscularly with 10 μg of algorithm-optimized H5 mRNA vaccine (eH5) or A/Jiangsu/NJ210/2023 (H5N1) mRNA vaccine (H5), with saline-injected mice as controls. Two doses were given at a 14-day interval. Sera were collected at 14 and 28 days after the prime immunization, and binding antibody levels were measured by ELISA. (A, B) Serum dilution-dependent IgG binding curves to A/Astrakhan/3212/2020 (H5N8) HA; (C) IgG antibody titers. (D, E) IgG1 binding curves; (F) IgG1 antibody titers. (G, H) IgG2a binding curves; (I) IgG2a antibody titers. (J, K) IgG2b binding curves; (L) IgG2b antibody titers. Data are shown as mean ± SD (n = 5). *$p < 0.05$, **$p < 0.01$, ***$p < 0.001$, ****$p < 0.0001$; ns, not significant.

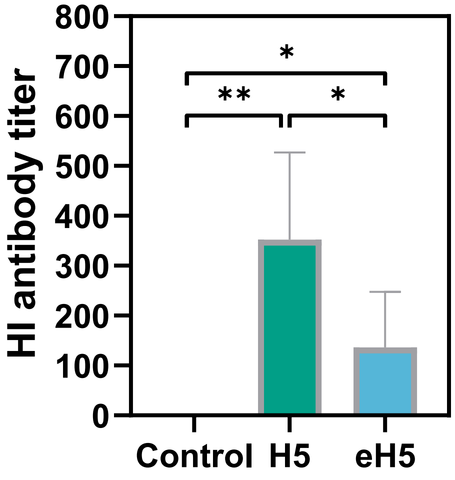

Figure S3. Hemagglutination inhibition antibodies against the inactivated A/Jiangsu/NJ210/2023 (H5N1) vaccine strain. Mice were immunized intramuscularly with 10 μg of algorithm-optimized H5 mRNA vaccine (eH5) or A/Jiangsu/NJ210/2023 (H5N1) mRNA vaccine (H5), with saline-injected mice as controls. Two doses were given at a 14-day interval. Sera were collected at 28 days after the prime immunization, and hemagglutination inhibition assay was performed to determine the titers of hemagglutination inhibition antibodies. Data are shown as mean ± SD (n = 5). $*p < 0.05$, $**p < 0.01$, $***p < 0.001$, $****p < 0.0001$; ns, not significant.
